# A motif-independent metric for DNA sequence specificity

**DOI:** 10.1186/1471-2105-12-408

**Published:** 2011-10-21

**Authors:** Luca Pinello, Giosuè Lo Bosco, Bret Hanlon, Guo-Cheng Yuan

**Affiliations:** 1 Department of Biostatistics, Harvard School of Public Health, 677 Huntington Avenue, Boston MA 02115, USA; 2 Department of Biostatistics and Computational Biology, Dana-Farber Cancer Institute, 44 Binney Street, Boston MA 02115, USA; 3 Dipartimento di Matematica ed Informatica, Via Archirafi 34, Palermo 90123, Italy; 4 Department of Statistics, University of Wisconsin, 1300 University Ave Madison, WI 53706, USA

## Abstract

**Background:**

Genome-wide mapping of protein-DNA interactions has been widely used to investigate biological functions of the genome. An important question is to what extent such interactions are regulated at the DNA sequence level. However, current investigation is hampered by the lack of computational methods for systematic evaluating sequence specificity.

**Results:**

We present a simple, unbiased quantitative measure for DNA sequence specificity called the Motif Independent Measure (MIM). By analyzing both simulated and real experimental data, we found that the MIM measure can be used to detect sequence specificity independent of presence of transcription factor (TF) binding motifs. We also found that the level of specificity associated with H3K4me1 target sequences is highly cell-type specific and highest in embryonic stem (ES) cells. We predicted H3K4me1 target sequences by using the N- score model and found that the prediction accuracy is indeed high in ES cells.The software to compute the MIM is freely available at: https://github.com/lucapinello/mim.

**Conclusions:**

Our method provides a unified framework for quantifying DNA sequence specificity and serves as a guide for development of sequence-based prediction models.

## Background

Of the entire 3GB human genome, only about 2% codes for proteins. The identification of biological functions of the entire genome remains a major challenge [1,2]. One powerful venue to gain functional insights is to identify the proteins that bind to each genomic region. Recent development of chromatin immunoprecipitation followed by microarray or sequencing (ChIP- chip or ChIPseq) technologies has made it feasible to map genome-wide protein-DNA interaction profiles [3-5]. The data generated by these experiments have not only greatly facilitated the genome-wide characterization of regulatory elements such as enhancers [6,7] but also been integrated with other data sources to build gene regulatory networks [8-11].

An important question is to what extent a specific protein-DNA interaction is mediated at the level of genomic sequences. While it is well known that specific sequence motifs are crucial for transcription factors (TF) mediated *cis*-regulation, there are many other proteins, such as chromatin modifiers, whose target sequences cannot simply be characterized by a handful of distinct motifs [12]. Such sequences are often regarded as nonspecific and not studied further. However, recent studies in nucleosome positioning have provided new insights by going beyond this motif-centric view [13]. Here various sequence features have been associated with nucleosome positioning, including poly dA:dT track [14,15], abundance of G/C content [16,17], and certain periodic patterns [18,19]. Such patterns cannot be captured by traditional motif analysis methods. Similar results have been obtained by analyzing histone modification [20,21] and DNA methylation data [22,23].

Despite the success of these recent sequence-based prediction models, it remains difficult to determine which sequences lack intrinsic specificity because a poor prediction outcome might imply than more sophisticated models. A guide is needed for developing sequence-based prediction models. To this end, here we present a simple approach to quantify sequence specificity based on the frequency distribution of *k*-mers. We will also systematically investigate the relative merit of various distance or similarity functions for capturing specific sequence information. While *k*-mers have been extensively to detect splice sites [24], to study functional genomic regions[25], to identify protein coding genes[26] and used in motif analysis (reviewed by [27]), to our knowledge, they have not been used to quantify sequence specificity.

We evaluated the performance of our approach by analyzing one simulated datasets and two real experimental datasets, corresponding to a TF (STAT1) and a histone modification (H3K4me1) respectively. Our results have provided new insights into the role of DNA sequences in modulating protein-DNA interactions regardless of motif presence.

## Results

### A simple measure of sequence specificity

While specific sequence information has been identified in the absence of distinct motifs, to our knowledge, it is always associated with enrichment of certain *k*-mers (where *k *is a small number, such as 4). Its main difference with motifs is that, when k is small, a single *k*-mer may occur many times in the genome and therefore would not be useful for any practical purpose. On the other hand, we reasoned that more specific information can be obtained by combinations of multiple *k*-mers. Therefore, it seems appropriate to quantify sequence specificity by aggregating enrichment information for all *k*-mers. For the rest of the paper, we fix *k *= 4, although the method presented below is equally applicable to any choice of k. Treating complementary sequences as identical, there are 136 non-redundant 4-mers. By counting the frequency of each 4-mer, each input sequence is then mapped to a 136 dimensional numerical vector containing the frequency of each *k*-mer. The distributions corresponding to sequences containing specific information should be distinct from those for random sequences, which are generated to match the number and length of the input sequences. We use the symmetric Kullback-Leibler (KL) divergence [28] for comparing frequency distributions and average over the entire set of input sequences. We term the resulting value as the *M*otif *I*ndependent *M*etric (MIM). To evaluate statistical significance, we estimate the null distribution by computing MIM values for sets of random sequences. The detailed procedure is described in the Methods section.

### Model Validation

#### Simulated data

As an initial evaluation, we synthetically generated 8 sequence sets each containing 2000 sequences, mimicking TF ChIPseq experiments for which the corresponding TF recognizes a single motif: **TTGACA**. The difference between these sequence sets is the motif strength, which is parameterized by a real number *ε *(see Methods). In particular, a perfect motif corresponds to *ε *= 0, whereas a random sequence corresponds to *ε *= 0.25. In a typical ChIPseq experiment, only a subset of target sequences contains the motif. To simulate this fact, we randomly selected 1000 sequences from each set and inserted the motif at a randomly selected location. As control, we also synthesized 1000 sets of 2000 random sequences each.

**Figure 1:**
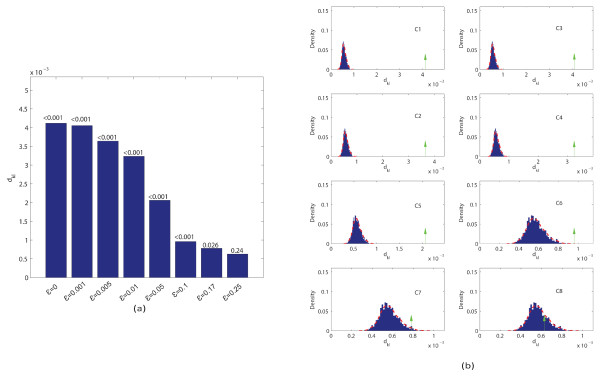
**MIM values for simulated sequences**. (a) The MIM values and corresponding p-values (above the bars) for the simulated data. Note that the MIM values change in the same direction as motif strength; (b) comparison of the MIM values with respect to the null distribution, which is obtained by using 1000 sets of random sequences.

We calculated the MIM values for each sequence set and evaluated the statistical significance of the resulting values. We found that the MIM values are statistically significant (p-value < 0.001) for *ε *up to 0.1 (Figure 1a and 1b). The information content for the corresponding motif is 5.35 bit, which is still lower than 98% of the motifs in the JASPAR core database [29]. In the following we will show that our method indeed performs well for real data. We ranked each *k*-mer according to its relative contribution to the MIM. The most informative *k*-mers are shown in Table 1. The methodology used to select such motif is outlined in the methods section. We noticed that the top *k*-mers are substrings of the inserted motif (highlighted in bold in Table 1), suggesting that these *k*-mers may be used as a seed for motif detection, in a similar way as the dictionary approach [30]. In additional to the KL divergence considered here, there are a number of other metrics to compare frequency distributions. We selected a few commonly used metrics and repeated the above analysis (Methods). We found that the results are quite similar (Table 2).

**Table 1:** Top 20 *k*-mers ranked by different distances on Cell1 of Synthetic dataset

	Cell1	
KL	Bhattacharyya	Hellinger
**tcaa**	**tcaa**	**tcaa**
**gaca**	**gaca**	**gaca**
**gtca**	**gtca**	**gtca**
acag	acag	acag
caag	caag	caag
attg	caac	attg
acat	acac	acat
caac	attg	caac
acac	acat	acac
aatg	aatg	aatg
acaa	acaa	acaa
ttaa	cgga	ttaa
aaat	caaa	aaat
cgga	gacc	cgga
caaa	agat	caaa
aatt	aaat	aatt
cata	taaa	cata
gacc	ccgc	gacc
agat	cgcc	agat
gtaa	aagg	gtaa
agat	cgcc	agat

**Table 2:** Distances values on Synthetic dataset

Cell	KL	p-value	Bhattacharyya	p-value	Hellinger	p-value
**1**	4.12E-03	<0.001	2.18E-03	<0.001	2.67E-02	<0.001
**2**	3.64E-03	<0.001	1.93E-03	<0.001	2.51E-02	<0.001
**3**	4.06E-03	<0.001	2.16E-03	<0.001	2.65E-02	<0.001
**4**	3.23E-03	<0.001	1.70E-03	<0.001	2.36E-02	<0.001
**5**	2.06E-03	<0.001	1.07E-03	<0.001	1.89E-02	<0.001
**6**	9.59E-04	<0.001	5.03E-04	<0.001	1.29E-02	<0.001
**7**	7.80E-04	0.0262	4.09E-04	0.0497	1.16E-02	0.0207
**8**	6.27E-04	0.2367	3.75E-04	0.1670	1.04E-02	0.2467

#### Real ChIPseq data

To validate our method using real experimental data, we analyzed a publicly available ChIPseq dataset for STAT1 [31], a member of the signal transducer and activator of transcription (STAT) family TFs, in the HeLa S3 cell line. The dataset contains 39,000 target sequences, 35% of which contains the consensus motif **TTCCNGGAA **(JASPAR database [29]). As control, we sampled random sequences from genomic background matching the number and length of the target sequences.

**Figure 2:**
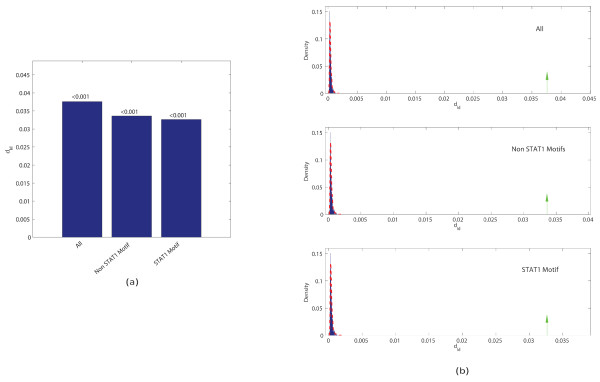
**MIM values for STAT1 target sequences**. (a) The MIM values and corresponding p-values (above the bars) for different subsets of STAT1 target sequences: all targets, STAT1 motif containing ones, and STAT1 motif absent ones; (b) comparison of the MIM values with respect to the null distribution, which is estimated by using 1000 sets of random sequences.

We evaluated the level of sequence specificity of the whole set of target sequences by using the MIM measure. The sequences are indeed highly specific (see Figure 2a and 2b). Again, among the top ranked *k*-mers, several are substrings of the "classic" STAT1 motif (highlighted in bold in Table 3), suggesting it may provide useful information for identifying discriminative sequence signatures without the knowledge of TF motifs. Furthermore, the results are not sensitive to the specific choice of distances as in the simulated data experiment (Table 4).

**Table 3:** Top 20 *k*-mers ranked by different distances on motif sequences on STAT1 dataset

	STAT1 Motif	
KL	Bhattacharyya	Hellinger
aata	atat	aata
ttaa	tata	ttaa
aaat	aata	aaat
aaaa	ttaa	aaaa
**ggaa**	atta	**ggaa**
atat	aaat	atat
atac	taaa	atac
tcaa	atac	tcaa
aatt	aatt	aatt
acat	ataa	acat
taca	taca	taca
aggg	cata	aggg
cgga	aaaa	cgga
atta	attg	atta
attg	acat	attg
taga	tcaa	taga
caaa	agcg	caaa
acta	gata	acta
ccag	taga	ccag
agca	cgga	agca

**Table 4:** Distances values on STAT1 dataset

Peaks	KL	p-value	Bhattacharyya	p-value	Hellinger	p-value
**Stat1 Motif**	3.26E-02	<0.001	2.19E-03	<0.001	7.51E-02	<0.001
**Non STAT1 Motif**	3.36E-02	<0.001	2.72E-03	<0.001	7.61E-02	<0.001
**All**	3.76E-02	<0.001	3.65E-03	<0.001	8.05E-02	<0.001

### Detecting sequence specificity in absence of a dominant motif

#### STAT1

As mentioned above, while the presence of STAT1 motif can explain the sequence specificity for 35% of the target sequences, it is unclear how TF is recruited to the other 65% of the targets. In order to evaluate the role of DNA sequence specificity for these motif-absent targets, we compared the MIM values between the motif-present and motif-absent subsets of targets. Surprisingly, we found that the MIM value for motif-absent targets is almost indistinguishable from motif-present targets (see Figure 2a and 2b). This high level of specificity cannot be simply explained by promoter-related biases, because only 11% of target sequences are located in promoters.

To gain mechanistic insights, we searched for enrichment of other TF motifs in the JASPAR database [29], using the FIMO software [32]. We found two motifs that are significantly enriched (threshold p-value < 10^-6^): SP1 and ESR1, both have previously been shown to interact with STAT1 [33,34]. Therefore, STAT1 might be recruited to the motif-absent targets through interaction with these other TFs. We further compared the associated gene ontology terms between the motif-present and motif-absent sets to see if there are any functional differences. We found that these two sets share many similar biological functions, such as hydrolase and ATPase activities (*p *< 10^-17^). On the other hand, while the motif-present targets are highly enriched for the voltage-gated calcium channel complex (*p *< 10^-12^), the motif-absent targets are highly enriched for cytoplasmic components instead (*p *< 10^-12^).

#### H3K4me1

**Figure 3:**
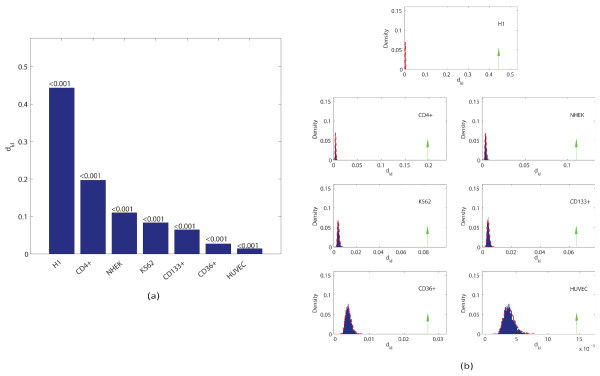
**MIM values for H3K4me1 target sequences**. (a) The MIM values and corresponding p-values (above the bars) for H3k4me1 target sequences in different cell lines. Note that the MIM value for H1 is much higher than for other cell lines; (b) comparison of the MIM values with respect to the null distribution, which is estimated from 1000 sets of random sequences.

Unlike TFs, histone (de)modifying enzymes usually do not directly interact with DNA. The role of DNA sequences in the regulation of histone modification patterns remains poorly understood. As an example, the histone modification H3K4me1 plays an important role in gene regulation by demarcating cell-type specific enhancers [6]; yet how it is recruited to enhancer regions is poorly understood. We hypothesized that the role of DNA sequence may play a cell-type specific role and aimed to detect such differences by using our MIM measure. To this end, we assembled an H3K4me1 ChIPseq dataset in seven human cell-lines, including H1 (a human embryonic stem cell line), K562 (a myelogenous leukemia cell line), Huvec (human umbilical vein endothelial cells), Nhek (normal human epidermal keratinocytes), and three T cell-lines (CD4+, CD36+, and CD133+) from the public domain [1,4,35]. For each cell line, we identified the peak locations by using cisGenome [36] then calculated the MIM value for DNA sequences at the peaks (in Table 5 the top 20 k-mers ranking by different distances on H1 cell line). The MIM values are highly cell-type specific (see Figure 3a and 3b and Table 6). Interestingly, the value for H1 cells is much higher than any other cell line, suggesting that the DNA sequence plays a unique role in H3K4me1 recruitment in ES cells. To eliminate the possibility that this difference may be simply due to a GC content related bias, we repeated the analysis by using a different null model, obtained by random shuffling the original sequences within each dataset. While the MIM values slightly change, they are ordered in nearly the same way as before (Additional File 1). Importantly, the MIM values are distinctively higher in the H1 cell line compared to the other cell lines, suggesting that such differences are unlikely due to a GC- content related bias.

**Table 5:** Top 20 *k*-mers ranking by different distances on H1 cell line on H3k4me1 dataset

H1 cell line		
KL	Bhattacharyya	Hellinger
tcga	tcga	tcga
cgaa	tcca	cgaa
attc	attc	attc
tcca	atgg	tcca
atcg	cgaa	atcg
ggaa	ggaa	ggaa
atgg	aatg	atgg
aatg	atcg	aatg
aacg	tata	aacg
ctta	ttaa	ctta
gcta	ctta	gcta
ttaa	aacg	ttaa
ctaa	gcta	ctaa
agct	taaa	agct
ggta	ggta	ggta
taaa	ataa	cgga
cgga	cgga	taaa
acgg	atta	acgg
ataa	acgg	ataa
atta	aaaa	atta

**Table 6:** Distances values on H3k4me1 dataset

Cell	KL	p-value	Bhattacharyya	p-value	Hellinger	p-value
**H1**	4.43E-01	<0.001	1.28E-02	<0.001	2.71E-01	<0.001
**Cd4+**	1.97E-01	<0.001	8.47E-03	<0.001	1.82E-01	<0.001
**NHEK**	1.10E-01	<0.001	4.10E-03	<0.001	1.37E-01	<0.001
**K562**	0.083176	<0.001	0.003584	<0.001	0.119491	<0.001
**Cd133+**	0.064867	<0.001	0.006796	<0.001	0.105424	<0.001
**Cd36+**	0.026875	<0.001	0.002996	<0.001	0.067992	<0.001
**HUVEC**	0.014557	<0.001	0.002912	<0.001	0.050102	<0.001

Since the H3K4me1 marks cell-type specific enhancers, one possible explanation for the high sequence specificity in ES cells is that the targets might be associated with a few ES-specific TFs. To test this possibility, we searched for enrichment of TF motifs in the JASPAR database using FIMO. Surprisingly, we were unable to find any significantly-enriched motif, suggesting that the specificity is contributed to a different mechanism.

**Figure 4:**
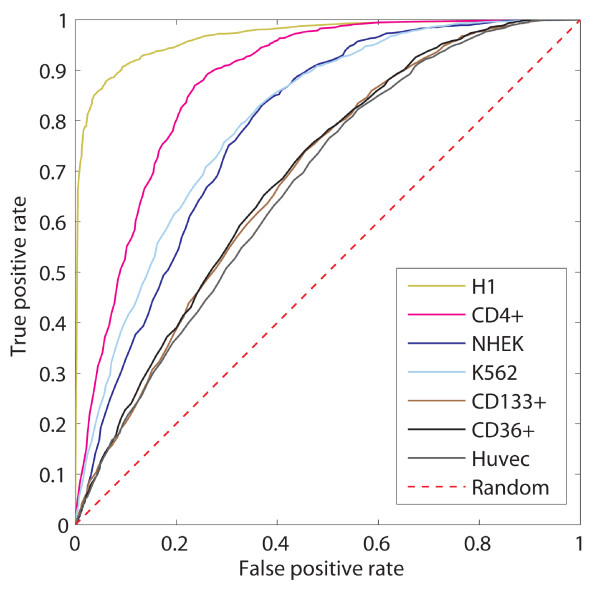
**N-score prediction of H3K4me1 target sequences**. Receiver operating characteristic (ROC) curves for different cell lines using the N-score. Note as the AUC for H1 is much higher than for other cell lines.

We then investigated whether the H3K4me1 targets in ES cells are indeed highly predictable. In previous work, we developed a sequence-based model, called the N-score model, to predict epigenetic targets [19,21]. This model integrates information from three classes of sequence features (sequence periodicity, word counts, and DNA structural parameters) by using stepwise logistic regression model (see methods for details). Here we applied the N-score model to predict H3K4me1 target sequences. As negative control, we selected the same number of sequences from the genome at random. We evaluated the model performance by using a 3-fold cross-validation. We found that prediction accuracy is indeed high for ES cells (AUC = 0.967) (Figure 4), whereas the accuracy for other cell types is much lower.

## Discussion

Recently it has been shown that a large number of proteins may weakly bind to DNA [37]. It remains unclear to what extent such events are mediated by specific sequence information. This question cannot be answered by using traditional motif analysis, since the target sequences do not contain distinct motifs. As an alternative approach, we define a simple measure, called MIM, to quantify sequence specificity by aggregating information from all *k*-mers. Our approach does not make any assumptions regarding motif presence, providing a more versatile tool for sequence analysis. We validated this method by analyzing both simulated and experimental data and found that it is indeed effective for detecting sequence specificity in both cases.

We also showed that the MIM measure can provide new biological insights. Specifically, we found that the motif-absent targets of a TF may also contain specific sequence information due to interaction with other TFs. We also found that the sequence specificity for H3K4me1 targets is higher in ES cells than in differentiated cell-types, suggesting a unique role of DNA sequence in the recruitment of H3K4me1 in ES cells. Interestingly, this high specificity cannot be explained by enrichment of known TF motifs, suggesting a yet uncharacterized recruitment mechanism in ES cells. The MIM algorithm is implemented in Python and can be freely accessed at : https://github.com/lucapinello/mim.

## Conclusion

The role of DNA sequence in gene regulation remains incompletely understood. Our MIM method has extended previous work by further accounting for sequence specificity due to accumulation of weak sequence features. The information can be used as a guide to systematically investigate the regulatory mechanisms for a wide variety of biological processes.

## Methods

### Synthetic data generation

We simulated ChIPseq data for a TF whose motif sequence is **TTGACA**. In order to simulate the variation of motif sites among different target sequences, we modeled the position weight matrix (PWM) as illustrated in Table 7, where ε measures the mutation rate of the motif and can change between 0 (perfect motif) and 0.25 (totally random). We sampled ε at 8 different values: 0, 0.001, 0.005, 0.01, 0.05, 0.1, 0.1667, and 0.25. For each choice of ε, we generated 2000 sequences of 500 bp each. The sequences were initially generated by randomly sampling from the background distribution with the probabilities of A,C,G,T equal to 0.15, 0.35, 0.35, 0.15, respectively. In addition, we randomly selected a subset of 1000 sequences and inserted the motif at a random location.

**Table 7:** PWM for synthetic motif generation

	1	2	3	4	5	6
**A**	ε	ε	ε	1-3ε	ε	1-3ε
**C**	ε	ε	ε	ε	1-3ε	ε
**G**	ε	ε	1-3ε	ε	ε	ε
**T**	1-3ε	1-3ε	ε	ε	ε	ε

### ChIPseq data source

Genome-wide STAT1 peak locations in HeLa S3 cell lines were obtained from the http://archive.gersteinlab.org/proj/PeakSeq/Scoring_ChIPSeq/Results/STAT1[31]. ChIPseq data for H3K4me1 in seven human cell lines were obtained from literature: CD4+ T cell [4], CD36+ and CD133+ T cells [35], H1, Huvec, K562, and Nhek [1]. The raw data were processed by cisGenome to identify peak locations [36]. The DNA sequences at the peak locations were analyzed subsequently.

### Motif analysis

Motif analysis was done by using several tools in the MEME suite (http://meme.nbcr.net/meme/) as follows. Scanning DNA sequences for matches of a known motif was done by using the FIMO [32]. Motif comparison was done by using TOMTOM.

### Functional annotation

Functional annotation was done by using the GOrilla software [38] (http://cbl-gorilla.cs.technion.ac.il/).

### Details of the MIM measure

Each DNA sequence is mapped to numerical values by enumerating the frequency of each *k*-mer treating complementary *k*-mers as the same. There are *m *= 136 non-redundant *k*-mers for k = 4. MIM is essentially a metric between two distributions of *k*-mer frequencies. Specifically, let **P = (***P*_*ij*_) be the *k*-mer frequency distributions corresponding to a set of *n *target sequences **S = (***S*_*i*_), where *S*_*i *_represents a sequence in the set **S**. We generate a set of *n *random sequences **R = *(****R*_*i*_) matching the sequence lengths (analogously *R*_*i *_represents a sequence in the set **R**). Let **Q **= (*Q*_*ij*_) be the *k*-mer frequency distributions corresponding to **R**. Finally let $P_{j}=\frac{\Sigma_{i}P_{ij}}{\Sigma_{ij}P_{ij}}$ and $Q_{j}=\frac{\Sigma_{i}Q_{ij}}{\Sigma_{ij}Q_{ij}}$ (**P**_***j ***_in particular represents the probability of the *j*-th *k*-mer in **S**, analogously, **Q**_***j ***_represents the probability of the *j*-th *k*-mer in **R**) then the difference between **P **and **Q **is quantified by the symmetrical Kullback-Leibler (KL) divergence [28], as follows:

$$d_{kl}\left(S,R\right)=\frac{\overset{m}{\underset{j=1}{\sum }}P_{j}log_{2}\frac{P_{j}}{Q_{j}}+\overset{m}{\underset{j=1}{\sum }}Q_{j}log_{2}\frac{Q_{j}}{P_{j}}}{2}$$

The MIM value corresponding to **S **is defined as the expected value *d*_*kl *_(**S**, **R**), which is estimated by averaging over 1000 sets of random sequences. The MIM value, using the symmetrical KL divergence, can be interpreted as the number of the expected number of extra bits required to code samples from **S **when using a code based on the background distribution. Note that there exist several alternatives to measure the similarity of two probability distributions [39]. To evaluate whether the results are sensitive to the specific choice of distances, we also computed MIM values based on two other well-known distances between probability distributions:

1) The Hellinger distance [39]

$$d_{hl}\left(S,R\right)=\sqrt{\frac{1}{2}\overset{m}{\underset{j=1}{\sum }}{\left(\sqrt{P_{j}-}\sqrt{Q_{j}}\right)}^{2}}$$

whose main differences from *d*_*kl *_are 1) *d*_*hl *_naturally satisfies the triangle inequality; and 2) the range of *d*_*hl *_is the interval [0,1].

2) The Bhattacharyya distance[40]

$$d_{bh}\left(S,R\right)=\overset{m}{\underset{j=1}{\sum }}\frac{{\left(\mu_{P_{j}}-\mu_{Q_{j}}\right)}^{2}}{4\left(\sigma_{P_{j}}+\sigma_{Q_{j}}\right)}+\frac{1}{2}\log\left(\frac{\sigma_{P_{j}}+\sigma_{Q_{j}}}{2\sqrt{\sigma_{P_{j}}+\sigma_{Q_{j}}}}\right)$$

which has been widely used for pattern recognition in computer science [41];

where $\mu_{P_{j}}=\frac{1}{n}\overset{n}{\underset{i=1}{\sum }}P_{ij}$ and $\sigma_{P_{j}}=\sqrt{\frac{1}{n-1}\overset{n}{\underset{i=1}{\sum }}{\left(P_{ij}-\mu_{P_{j}}\right)}^{2}}$ are the mean and standard deviation, respectively, of **P**_***j ***_($\mu_{Q_{j}}$ and $\sigma_{Q_{j}}$ are defined similarly for **Q**_***j***_).

In order to estimate the null distribution, we generated 1000 sets of random sequences and then calculated MIM values for each random sequence set. The probability density function (pdf) was estimated by using a kernel method [42]. This pdf was used to infer not only the mean and standard deviation of the null distribution but also the statistical significance for any MIM value. Recognizing the limited resolution of the estimated pdf, we did not distinguish p-values that are smaller than 0.001.

### N-score model

The N-score model was described previously [19,21]. In brief, the model integrates three types of sequence features, including sequence periodicities [19], word counts [16], and structural parameters [43], a total of 2920 candidate features. Model selection was done by stepwise logistic regression. The final model was used for target prediction.

### Most informative k-mers selection

Giving **P**_***j ***_and **Q**_***j ***_associated to **S **and **R **respectively, it is possible to calculate their Kullback-Leibler (KL) divergence for each *j*, where *j *indicates the *j*-th *k*-mer component. This results in a list of 136 distance values, whose ranking can be used as a guide to identify the most informative *k*-mers.

## Authors' contributions

LP and GY conceived and designed the study. LP and GL have implemented the MIM methodology. LP and BH analyzed the data. LP and GY interpreted the data. All authors wrote, read and approved the manuscript.

## Supplementary Material

Additional file 1 **Choice of the null model for sequence specificity**. (a) The MIM values for H3k4me1 target sequences in different cell lines experiment with a null model obtained shuffling the original sequences. (b) The MIM values for the same experiment using as a null model a set of random sequences extracted from genome with matching lengths. Note that the the H1 cell line is far more specific than the other cell lines independently of the null model chosen. Click here for file
