# Supplementary figures and images for: A motif-independent metric for DNA sequence specificity
Source: BMC Bioinformatics. 2011 Oct 21;12:408. doi: 10.1186/1471-2105-12-408 (PMC3267244; doi:10.1186/1471-2105-12-408)

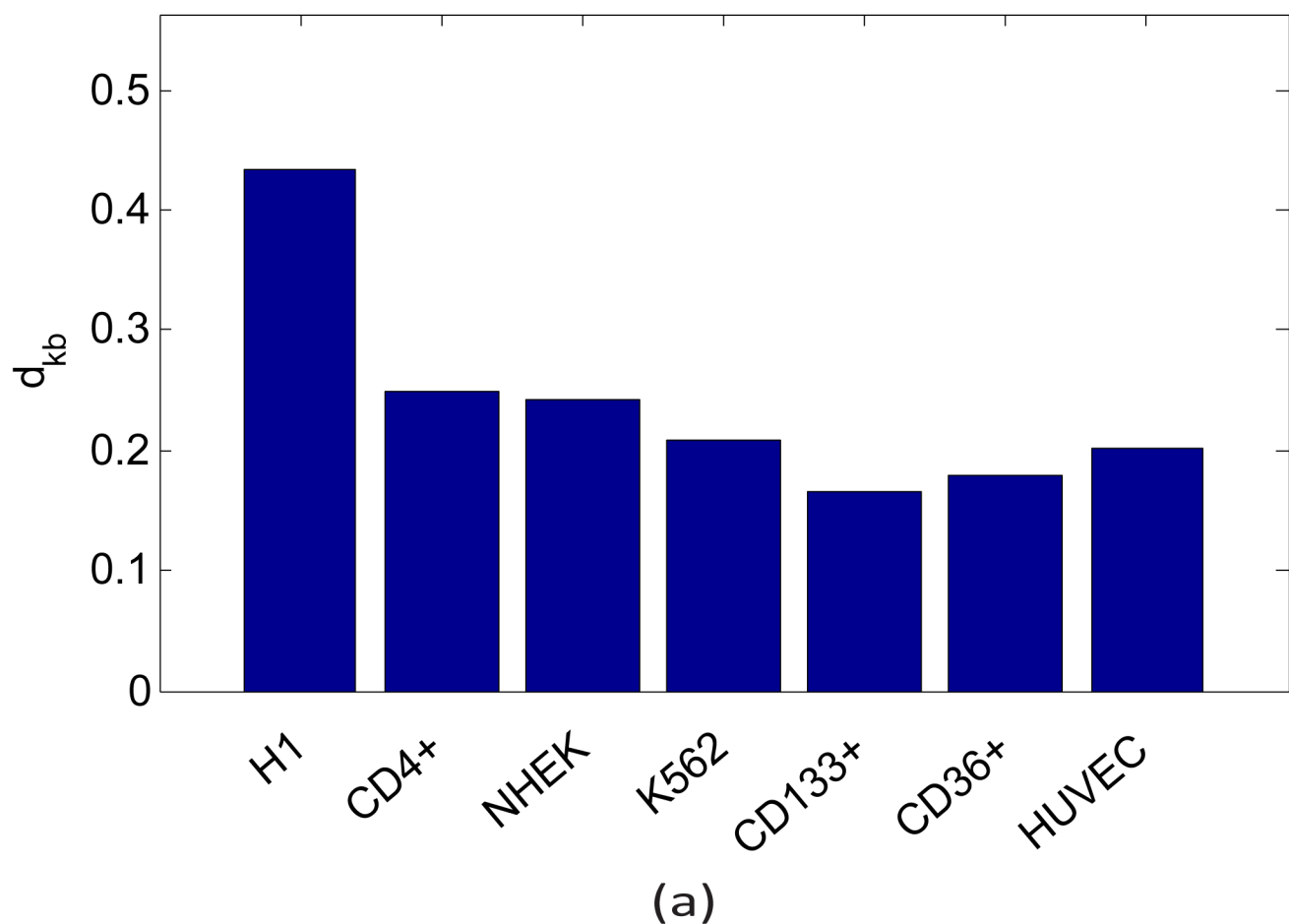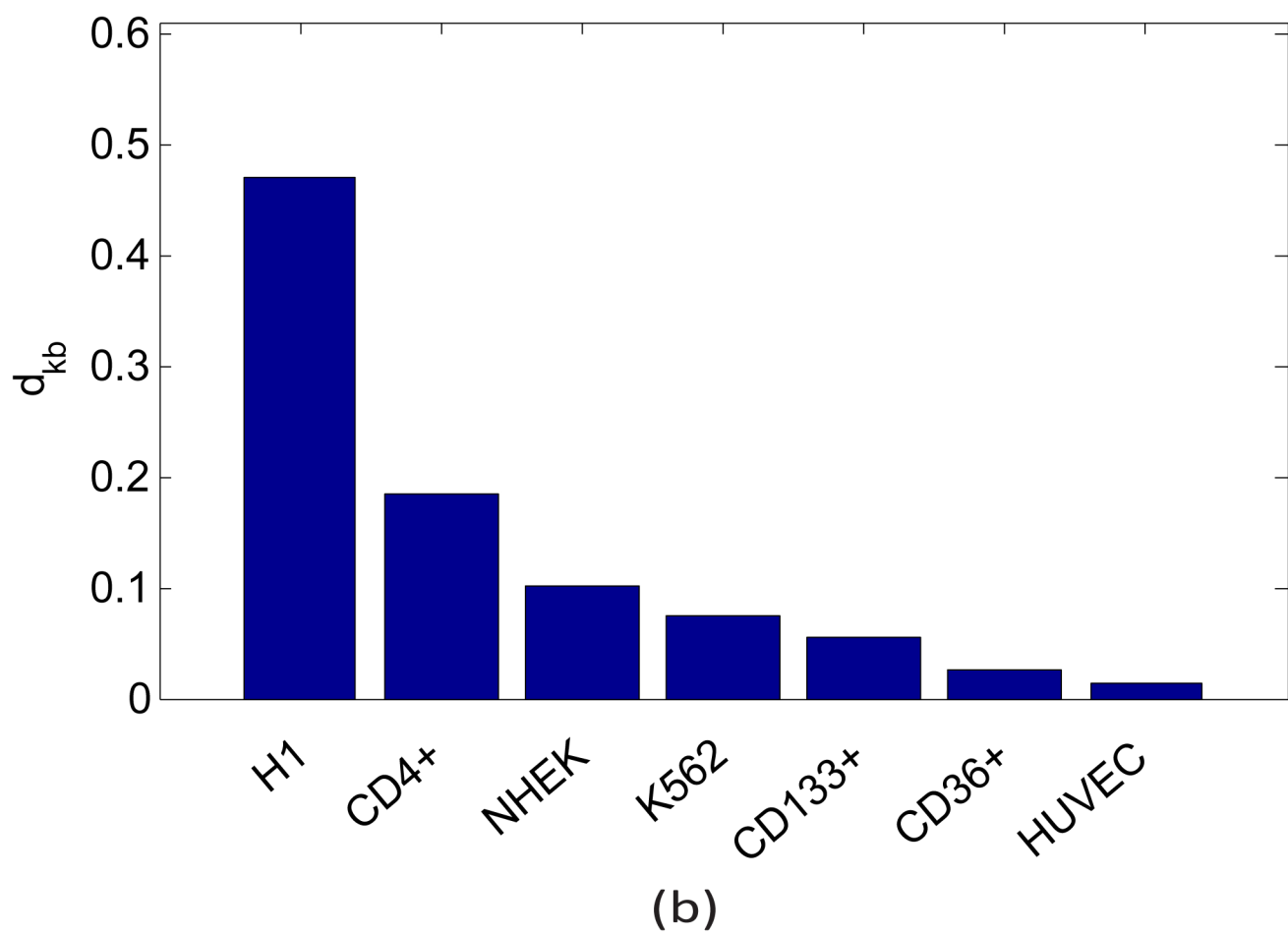

Supplement: Additional file 1 — Choice of the null model for sequence specificity. (a) The MIM values for H3k4me1 target sequences in different cell lines experiment with a null model obtained shuffling the original sequences. (b) The MIM values for the same experiment using as a null model a set of random sequences extracted from genome with matching lengths. Note that the the H1 cell line is far more specific than the other cell lines independently of the null model chosen. [file 1471-2105-12-408-S1.PDF]
